# Ethnobotanical Study on Medicinal Plants Used by the Local Communities of Ameya District, Oromia Regional State, Ethiopia

**DOI:** 10.1155/2023/5961067

**Published:** 2023-12-01

**Authors:** Temesgen Tadesse, Alemtshay Teka

**Affiliations:** ^1^Department of Biology, College of Natural and Computational Sciences, Kotebe Metropolitan University, Addis Ababa, Ethiopia; ^2^Endod and Other Medicinal Plants Research Unit, Aklilu Lemma Institute of Pathobiology, Addis Ababa University, Addis Ababa, Ethiopia

## Abstract

In the present study, the diversity of medicinal plants and associated traditional medicinal knowledge of the rural community in the Ameya district in Ethiopia was assessed and documented. A survey was conducted through semistructured interviews, guided field walks, focus group discussions, and field observations. The snowball and purposeful sampling techniques were employed to select general and knowledgeable informants, respectively. Accordingly, a total of 210 respondents, 156 (74.3%) males and 54 (25.7%) females, were participated in this study. The informants were selected from seven kebeles (the lowest administrative units) following the recommendations of the local community for the availability of traditional medicinal plant use practice. Descriptive statistics, preference ranking, fidelity level, informant consensus factor, and direct matrix ranking were used to analyze and present the data. A total of 78 medicinal plants represented in 70 genera and 40 families were identified. *Croton macrostachyus* and *Dodonaea viscosa* were the most preferred species used to treat wounds, whereas *Cucumis ficifolius* and *Phragmanthera macrosolen* were the most popular species applied to treat stomachache. Leaves (38%) and roots (20%) were the most predominantly used plant parts for remedial preparation to treat 42 human ailments. The informant consensus factor (ICF) value ranged between 0.45 and 0.81, with the respiratory diseases category scoring the highest ICF value. The fidelity level (FL) value for the medicinal plants ranged from 24 to 95%. Considerable proportions (55.5%) of the medicinal plants were collected from wild habitats. Higher ICF (0.81) and FL (>90%) scores indicate the presence of rich traditional knowledge in the community. This knowledge can be used to select medicinal plants (such as *Croton macrostachyus*, *Cucumis ficifolius*, *Dodonaea viscosa*, and *Phragmanthera macrosolen*) for further pharmacological and phytochemical studies.

## 1. Introduction

Since time immemorial, traditional medicine has been used in healthcare systems. Consequently, in many developing countries, 80% or more of the population mostly living in rural areas relies on traditional medicine to ensure their primary healthcare needs [1]. There is also a rising trend in the application of complementary and alternative medicine of herbal origins in developed countries [2]. In Ethiopia, approximately 80% of humans and 90% of the livestock population depend on traditional medicine which is largely plant-based [1, 3]. The long history and higher percentage of using traditional medicinal plants were attributed to the cultural acceptability, easy access, proven effectiveness by the community, and low cost of traditional medicine [4–6]. Recently, research undertaken on traditional medicinal plants has caught scientific interest. Moreover, the potential of medicinal plants as a source of new drugs is increasingly recognized [7–9]. The role of plant-derived antimicrobials is gaining acknowledgment as the future strategy for treating some incurable infections [10, 11].

In spite of the significant contribution of traditional medicine in primary health care and its potential, the system has faced problems of continuity and sustainability. In Ethiopia, probably in other countries, medicinal plants are getting lost mainly due to anthropogenic factors such as over-harvesting, increased population pressure, agricultural expansion, firewood collection, and charcoal production [12–15]. Due to these, the healers and the local community have witnessed the difficulty of finding some medicinal plants through time. Consequently, they walk unusually far from their vicinity in order to collect the medicinal plants. These might lead to the ultimate extinction of medicinal plants especially those that are known to be locally rare and most widely used by the community. On top of this, associated indigenous knowledge is also at verge of loss as the younger generation is lacking interest on traditional medicine. Such incidences were frequently reported in Ethiopia and elsewhere [16–19]. Thus, knowledgeable elders have received less attention and may pass away without conveying their knowledge to the next generation.

Considering the diverse agroecologies, Ethiopia has a huge potential in discovering new plant-based medicine. Similarly, medicinal plants contribute significantly to rural livelihoods in the Ameya district. Thus, enduring and optimizing the benefits of medicinal plants require documenting the traditional medicinal plants' wealth. However, published data on the diversity of medicinal plants and associated indigenous knowledge in the Ameya district are lacking. Rather, the medicinal plants and associated indigenous knowledge in Ethiopia, particularly in the Ameya district, are under pressure due to several anthropogenic factors such as deforestation and agricultural expansion. These call for proper documentation of associated indigenous knowledge, development of the medicinal plant database of the country, and integrative conservation measures of medicinal plants. Therefore, this study was intended to identify medicinal plant diversity, document associated indigenous knowledge of the local people, identify the most important medicinal plants, and pinpoint local threats to the plants in the Ameya district, Ethiopia. Finally, the study would contribute towards the development of the country's medicinal plants database and recommend candidate plants for further laboratory analysis.

## 2. Materials and Methods

### 2.1. Description of the Study Area

**Figure 1:**
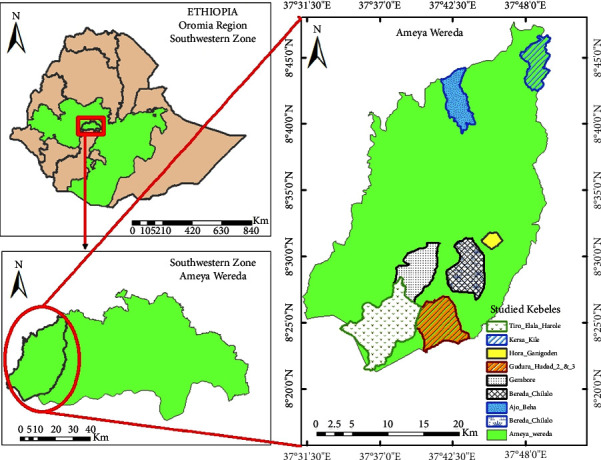
Map of the study area, Ameya district, Ethiopia.

Ameya district is found in South West Shewa Zone, Oromia Regional State, Ethiopia. The district is located between 8°21.5′ to 8°47.5′N and 37°31′ to 37°50′E (Figure 1). Gindo is the administrative town of the district. It is located 145 kms away from the capital city of Ethiopia. According to Ameya District Rural and Development Bureau report [20], there are 36 kebeles (the lowest administrative units) in the district. The altitude of the district ranges between 1600 m and 2490 m.a.s.l. It is characterized by hilly (mountainous), undulating, and dominantly flat landforms. The majority of land (60%) is covered by cultivated land. The district is classified into three agroecological zones: lowland (locally called Gammoojjii) 40%, midland (Baddadaree) 36%, and highland (Baddaa) 17%. The area has a minimum of 900 mm and a maximum of 1600 mm of rainfall per year, and its annual temperature ranges from 15°C to 28.4°C [20, 21]. The total population of the district was 112,794, of whom 55,551 were males and 57,243 females. There were 16,200 households of which 15876 were male-led and 324 female-led. Out of the total population, 99% live in rural areas, and the rest 1% live in town [22].

### 2.2. Study Sites and Informant Selection

A total of seven study sites or kebeles were purposively selected based on the recommendation of the local community and knowledgeable elders. These include Bereda chilalo, Gembore, Kersa Kile, Gudura Hudad 2&3, Tiro Elala Harole, Hora Ganigoden and Ajobeha. A simple random snowball sampling technique was employed to select 210 informants (156 male and 54 female) [23]. The informant's age range is between 18 and 90 years old. Based on the recommendation of the local community, 11 key informants were purposefully selected. Focus group discussion with the key informants was undertaken four times in order to verify information obtained through individual interviews and identify existing threats to medicinal plants [24].

### 2.3. Ethnobotanical Data Collection

Ethnobotanical field data collection was conducted from September 2020 to January 2021. The study sites were visited three times. Semistructured interviews, guided field walks, observation, and focus group discussion were employed following standard ethnobotanical methods [23, 25, 26]. The data collection was done based on a checklist of questions prepared in English and translated into two local languages “Afan Oromo” and “Amharic.” Data related to the informant's characteristics (sex, age, and educational level), ailments treated using medicinal plants, preparation methods, routes of administration, threats of medicinal plants, conservation practices, and other uses of medicinal plants were collected. The marketability of the reported medicinal plant was assessed in three local markets (Gindoo, Berema, and Bereda) of the study area.

### 2.4. Plant Specimen Collection and Identification

Specimens of all reported medicinal plants were collected during guided field walks with the help of key informants, pressed, and dried for identification. Preliminary identification of the plants was carried out with the help of local experts and published plant taxonomy keys and illustrations. Further identification of the specimens was carried out using taxonomic keys in the Flora of Ethiopia and Eritrea Volumes [27–31] and in comparison with authentic specimens. Later, Dr. Ermias Lulekal (Associate Professor) verified the identification. The voucher specimens were labeled and deposited in the National Herbarium (ETH), Addis Ababa University, Ethiopia.

### 2.5. Data Analysis

Descriptive statistics (such as proportions and frequency of citation) were used to analyze data related to methods of preparation, routes of administration, disease treated, plant parts used, and growth forms of the medicinal plants. Pictorial representation and numerical variables of the survey data were also presented by using graphs and tables.

The most preferred medicinal plant used to treat stomachache and the most threatening factor to the medicinal plants in the study area were identified using the ranking method [23, 26, 32]. Preference ranking was computed to assess the degree of effectiveness of six medicinal plants, which were reported to cure stomachache. This ailment was among the most commonly occurring diseases in the study area, and it is usually treated by using herbal remedies. To score the most preferred medicinal plants, eleven key informants were provided with the leaves of six medicinal plants along with their respective paper-tagged local name. The key informants were asked to assign the highest value “6” for the most effective medicinal plant against stomachache and the least value “1” for the least effective plant. The scores were summed up, and ranks were given to each plant species. Similarly, the most threatening factors to the medicinal plants were identified using a priority ranking of the frequently stated six threatening factors in the study area.

Direct matrix ranking was computed to identify the best multipurpose medicinal plant species in the study area [23, 33]. Eleven key informants were asked to assign values to the seven most cited multipurpose medicinal plants in six use categories (medicine, furniture makings, charcoal production, firewood, construction, and fencing). The key informants assigned “6” to the most used plants for a particular use category and “1” to the plant used the least to the use category stated. Finally, the values were summed, and the plant use for each category was ranked.

The paired comparison was computed to evaluate the degree of preference or level of importance of six selected medicinal plants, which were reported as effective for treating wound [23, 25, 34]. This ailment was frequently reported as being treated using herbal remedies. Eleven key informants were asked to compare the given paired medicinal plants based on their efficacy. The sequence of the pairs and the order within each pair were randomized and presented to the key informants. The responses were recorded, and total scores were summed and ranked. Rank was calculated by counting the number of times a medicinal plant was selected from the list of pairs provided. All possible combinations (15) of the plants were computed using the formula *n* (*n* − 1)/2, *n* = the number of medicinal plants being compared.

Fidelity level (FL) reflects the preference of people for a specific medicinal plant species for a particular illness [32, 34, 35]. It was calculated using the following equation: FL (%) = Np/*N* × 100, where Np is the number of informants who mentioned or claimed the use of plant species for a particular medicinal treatment and *N* = is the total number of informants who cited the plant species for various kinds of medicinal use.

Informant consensus factor (ICF) was calculated to determine the homogeneity of the information for a particular plant to treat a particular ailment category [19, 33, 36]. Reported ailments were categorized based on the body part affected. ICF values indicate the presence of potentially effective medicinal plant species within the respective disease categories. To determine the informant consensus factors, all the recorded 42 ailments were grouped into 8 major use categories. It was calculated using the following formula described in [25]. ICF = (*N*_ur_ − *N*_*t*_)/(*N*_ur_ − 1), where “*N*_ur_”refers to the total number of use reports for a particular illness category and “*N*_*t*_” refers to the total number of species used for this illness category.

ICF values range from 0 to 1. A high ICF value (close to 1) of an ailment category is obtained when one or a few plant species are documented to be used for the treatment of the category by a large proportion of the informants. A higher informant consensus value suggests that the informants have a better agreement on the use of a certain species for the treatment of a particular ailment category, whereas a low ICF (close to 0) value indicates that informants disagree over which plant to use for the use category stated or there is no exchange of information about their use among informants [37].

## 3. Results

### 3.1. Respondents' Character and Indigenous Knowledge Transfer

A total of 210 respondents, 156 (74.3%) males and 54 (25.7%) females, participated in this study. The age of the respondents ranged from 18 to 90. Most (48%) of the participants were in the age range of 31–56 years of age, 30.5% were above 56 years of age, and the remaining 21.5% were below 30 years of age. Concerning the educational level of the participants, 70% were illiterate and 30% were literate. Most (83%) of the respondents acquired medicinal plants use knowledge through interaction with neighbors/relatives, 10% of the informants learned the knowledge from traditional healers, and the remaining 7% learned through reading books.

### 3.2. Medicinal Plant Diversity, Growth Form, and Habitats

**Figure 2:**
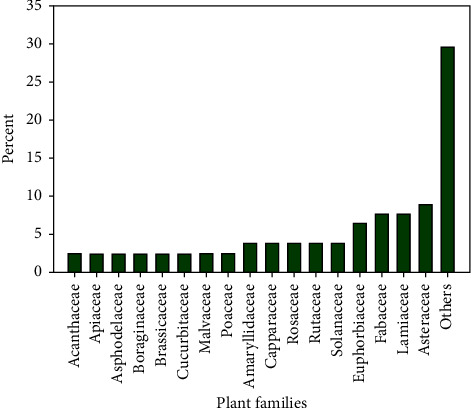
Proportion of plant species across plant families.

**Figure 3:**
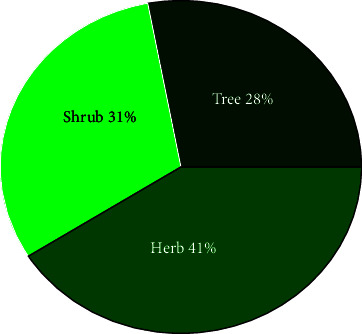
Percentage of the growth forms of medicinal plants used in the study area.

A total of 78 medicinal plant species, belonging to 70 genera and 40 families, used by the local community to treat various human and livestock diseases were documented (Additional File 1). Asteraceae (9%) and Fabaceae (8%) were the most represented plant families (Figure 2). Twenty-nine plant families were represented by a single species. Among the total documented medicinal plant species, 20 plant species (25%) (represented in 20 genera and 15 families) were reported to treat only livestock ailments. Herbs constituted the highest proportion (32 species, 41%) of the medicinal plant's growth form followed by shrubs (24 species, 30%) and trees (22 species, 28%) (Figure 3). Higher proportions (55.5%) of the medicinal plants were obtained from the wild habitat. The remaining were collected from homegardens (30.8%), and 13.5% of the medicinal plants were collected from both homegardens and wild habitats.

### 3.3. Medicinal Plant Parts Used

**Figure 4:**
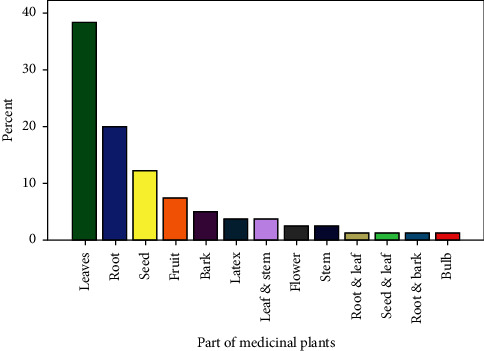
Plant parts are used for the preparation of remedies.

Leaves were the most predominantly (31 spp., 38%) used medicinal plant part followed by roots 16 (20%), seeds 10 (12%), fruits 6 (7%), and bark 4 (5%) (Figure 4).

### 3.4. Mode of Remedial Preparation and Routes of Administration

**Figure 5:**
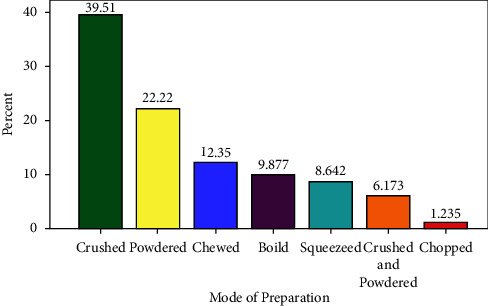
Mode of preparation of medicinal plants.

The medicinal plants for the treatment of human and livestock ailments were prepared using seven different methods. Crushing the plant parts was the main mode of preparation 32 (44.4%), followed by powdering 18 (25%) and chewing 10 (13.9%) (Figure 5). Most (80%) of the remedy preparation involved fresh forms rather than dry or a combination of fresh and dry forms (20%).

**Figure 6:**
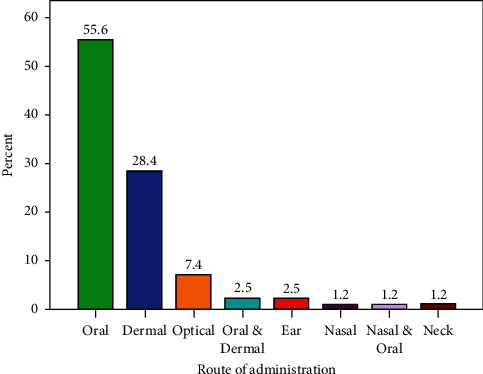
Routes of remedial administration.

Oral administration 45 (56%) was the main route of remedy administration in the study area followed by dermal 23 (28%) and optical 6 (7%) applications (Figure 6). The dosage of the administered herbal remedies depends on the experience of the person who prepares the remedy, the age of the patient, or the severity of the illness.

### 3.5. Effective Medicinal Plants

The preference ranking of six medicinal plant species used for treating stomachache was assessed. *Cucumis ficifolius* ranked first indicating that it is the most effective in treating stomachache followed by *Phragmanthera macrosolen* and the least preferred species was *Citrus aurantium* (Table 1). The paired comparison showed that *Croton macrostachyus* ranked first indicating that it is the most effective remedy for treating wounds followed by *Dodonaea viscosa* (Table 2), whereas *Prunus africana* and *Coffea arabica* were the least preferred for wounds compared to the others provided for ranking.

### 3.6. Fidelity Level Index

The fidelity level of eight frequently cited medicinal plant species ranges from 24% to 95% (Table 3). *Eucalyptus globulus* demonstrated the highest FL for cough (95%), followed by *Croton macrostachyus* (92%) and *Gymnanthemum auriculiferum* (90%) for treating wounds and malaria, respectively. The least scored (24%) FL was for *Artemisia abyssinica* used for treating evil spirit.

### 3.7. Informant Consensus Factor

Informant consensus factor (ICF) ranged from 0.45 to 0.81 (Table 4). The results showed that the respiratory tract disease scored the highest ICF value (0.81).

### 3.8. Direct Matric Ranking

Direct matrix computed value showed that *Eucalyptus globulus* was the most multipurpose medicinal plant followed by *Cordia africana* and *Hagenia abyssinica*, respectively (Table 5).

### 3.9. Marketability of Medicinal Plant

Some medicinal plants were sold in the local markets for their medicinal and other uses (Table 6). Plants such as *Allium sativum*, *Coffea arabica*, *Nigella sativa*, and *Zingiber officinale* were widely sold in the market for their nonmedicinal values mainly as a source of food and/or as flavoring foods and beverages.

### 3.10. Threats to Medicinal Plants

The medicinal plant species and associated knowledge are getting lost due to anthropogenic factors such as drought, deforestation, agricultural expansion, construction, and firewood consumption. In the present study, it was reported that agricultural expansion and overgrazing ranked as the 1st and 2nd top causes of medicinal plant loss in the area (Table 7).

## 4. Discussion

Few families (Asteraceae and Fabaceae) were highly represented in the present study. Similarly, other ethnobotanical studies conducted in different parts of the country reported the dominancy of these families in the respective study areas [17, 34, 38, 39]. The higher number of species represented in Asteraceae and Fabaceae families could be attributed to the wider distribution and abundance of these families and associated knowledge in the Flora regions of Ethiopia [27, 28, 40].

Herbs were the most reported plant species in the study area. This might be due to the easy availability of the plants in the area and abundant associated knowledge among the community. Similarly, most ethnobotanical studies in different parts of Ethiopia reported the dominance of herbs in traditional medicine [13, 17, 34]. Regarding the source of medicinal plant collection, considerable proportions of the medicinal plants were collected from the wild habitats. This makes wild habitats an important source of herbal medicine for rural communities. However, the collection of medicinal plants only from forests might create pressure on the survival of medicinal plants especially for the most sought medicinal plants [34, 41]. Thus, the practice of cultivating the plants needed to be enhanced.

Leaves were the most dominant medicinal plant parts used in the present study area and elsewhere in the country [19, 34, 42, 43]. The use of leaves as compared to roots is considered less destructive especially to trees and shrubs [43, 44]. Crushing the plant part was the main mode of preparation of remedy in the study area as reported in other studies conducted elsewhere in the country [43, 45]. With respect to the routes of remedy administration, the local community recommends different routes depending on the type of disease treated. Lack of endorsement on the type and dosage of herbal remedies to be administered is considered as one of the drawbacks of traditional herbal remedies application in the study area and elsewhere [17, 19]. Vomiting, diarrhea, nausea, and stomach burning were the most frequently reported side effects or adverse reactions that may be experienced after taking the dose. Possibilities of experiencing similar side effects from some medicinal plants are also a common complaint reported in different studies [17, 33].

Medicinal plants that are widely known to treat a single or few ailments score higher fidelity levels. In the present study, *Eucalyptus globulus* (95%), *Croton macrostachyus* (92%), and *Gymnanthemum auriculiferum* (90%) scored the highest FL value. Those species that scored lower FL value such as *Artemisia abyssinica* (24%) might explain that the species are less abundant in the study area or there is little information about the use of this medicinal plant among the people of Ameya district. However, it is possible that the plant might be used for treating many ailments. Similar scenarios were reported in studies conducted in different parts of the county [14, 46]. Due to these reasons, plants that scored low FL should not be disregarded from further laboratory analysis or conservation efforts to be undertaken, whereas higher ICF value indicates that informants use relatively few taxa to manage the disease conditions, and there is uniformity in the use of plant species for the given use category. In the present study, the respiratory tract disease scored the highest ICF value (0.81). This might indicate the fact that there are many traditional medicinal plants commonly known to treat respiratory tract diseases that are popular and considered effective by the local community. It may also imply that respiratory tract problems are frequently occurring diseases in the area. In contrast, the low value of ICF indicates that the informants disagree on the taxa to be used for treating the stated disease category [33, 46].

The medicinal plants sold in the local markets were primarily used for other purposes than their medicinal value. This might indicate that the local community either freely collects most of the medicinal plants from their natural habitat or obtains the remedies from healers [47]. This could cause pressure on the sustainability of plants due to overexploitation in the study area especially for the most frequently sought medicinal plants [45].

The most common (83%) means of acquiring traditional knowledge in the study area were from neighbors/relatives. This could reflect the fact that the practice of traditional knowledge transfer from the healers is very low. Rather, the existing knowledge within the community was developed through interaction mainly with neighbors, relatives, or friends. Thus, it can be presumed that very important and unique traditional healer's knowledge is conserved among them and might be lost without being properly documented.

Agricultural expansion, overgrazing, and drought were reported as the primary causes of plant loss in the study area. Similar threats to medicinal plants were reported in various studies conducted in Ethiopia and elsewhere [19, 47–49]. The presence of some practice of controlled harvest from the natural forest was reported. It limits the harvesting period or else collecting plants that are considered spiritually or culturally important is completely forbidden. As a result, traditionally protected areas usually contain diversified medicinal plant. Thus, such areas could be considered as priority sites for the protection of medicinal plants and the search for new candidate plants for further pharmacological studies.

## 5. Conclusions

The study area has diverse flora and rich traditional medicinal plant knowledge used for the treatment of various human and animal ailments. Higher percentages of medicinal plants are freely accessed from their natural habitat, which calls for the initiation of conservation practice including promoting their cultivation in homegardens. In order to optimize the benefits obtained from traditional medicinal plants, undertaking further pharmacological and phytochemical studies on culturally preferred plants (such as *Croton macrostachyus*, *Cucumis ficifolius*, *Dodonaea viscosa*, and *Phragmanthera macrosolen*) is recommended.

## Figures and Tables

**Table 1:** Preference ranking of seven medicinal plants used to treat stomachache.

Medicinal plant species	Respondents (*R*_1_–*R*_11_)											Total	Rank
	*R*_1_	*R*_2_	*R*_3_	*R*_4_	*R*_5_	*R*_6_	*R*_7_	*R*_8_	*R*_9_	*R*_10_	*R*_11_		
*Cucumis ficifolius*	5	5	4	5	6	6	4	6	6	5	6	58	1^st^
*Phragmanthera macrosolen*	5	4	5	4	5	6	4	5	5	6	4	53	2^nd^
*Citrus limon*	5	3	4	3	6	4	3	4	3	4	5	44	3^rd^
*Ruta chalepensis*	4	3	3	4	4	3	5	2	3	4	5	40	4^th^
*Capparis sepiaria*	6	4	2	2	3	4	3	2	3	3	4	36	5^th^
*Calpurnia aurea*	3	5	4	4	2	3	2	2	1	3	2	31	6^th^

**Table 2:** Paired comparisons of six medicinal plants used to treat wounds.

Medicinal plant species	Respondents (*R*_1_–*R*_11_)											Total	Rank
	*R*_1_	*R*_2_	*R*_3_	*R*_4_	*R*_5_	*R*_6_	*R*_7_	*R*_8_	*R*_9_	*R*_10_	*R*_11_		
*Croton macrostachyus*	5	4	3	5	3	4	4	5	3	5	4	45	1^st^
*Dodonaea viscosa*	3	5	3	4	2	4	3	4	2	4	4	38	2^nd^
*Gymnanthemum auriculiferum*	3	2	4	2	3	3	4	2	3	3	3	32	3^rd^
*Aloe* sp.	2	2	2	0	2	1	2	1	2	2	2	18	4^th^
*Prunus africana*	1	2	1	2	3	1	1	2	2	1	1	17	5^th^
*Coffea arabica*	1	0	2	2	2	2	1	1	3	0	1	15	6^th^

**Table 3:** Fidelity level values of medicinal plants against most frequently treated diseases.

Medicinal plant species	Ailment treated	NP	*N*	FL%
*Eucalyptus globulus*	Cough	194	204	95
*Croton macrostachyus*	Wound	108	117	92
*Gymnanthemum auriculiferum*.	Malaria	63	70	90
*Buddleja polystachya*	Eye infection	21	24	88
*Cucumis ficifolius*	Stomach ache	74	86	86
*Ehretia cymosa*	Toothache	19	25	76
*Euphorbia tirucalli*	Itchy	10	14	71
*Artemisia abyssinica*	Evil sprit	8	33	24

**Table 4:** Informant consensus factor (ICF) is the major use category reported.

Disease category	*N*_*t*_	*N*_ur_	ICF
Respiratory diseases	8	38	0.81
Animal diseases	4	15	0.78
Emergency diseases^*∗*^	5	15	0.71
Gastrointestinal problems	15	36	0.6
Organ and system diseases	28	68	0.59
Dermatological diseases	19	34	0.45

“^*∗*^Emergency diseases, suddenly feeling ill, of unknown cause also locally called “Mich.”

**Table 5:** Direct matrix ranking of seven multipurpose species.

Medicinal plant species	Use categories						Total	Rank
	Medicine	Furniture	Charcoal	Firewood	Construction	Fencing		
*Eucalyptus globulus*	12	6	6	23	21	24	92	1^st^
*Juniperus procera*	3	24	1	6	20	6	60	6^th^
*Senegalia senegal*	3	17	22	15	10	4	71	4^th^
*Ficus sycomorus*	2	14	16	12	17	6	67	5^th^
*Afrocarpus falcatus*	3	18	13	12	4	5	55	7^th^
*Hagenia abyssinica*	22	23	1	6	24	12	88	3^rd^
*Cordia africana*	13	20	12	14	20	13	91	2^nd^
Total	58	122	71	88	116	70		

**Table 6:** List of medicinal plants widely sold in the market.

No.	Scientific name	Local name (Amharic)	Used for	Unit price birr^*∗*^/Measurement
1	*Allium sativum*	Qullubbiiadii	Medicinal, food, spices	200 birr/kg
2	*Aloe* sp.	Eret	Medicinal, edible	30 birr/pieces
3	*Carica papaya*	Paappaayyaa	Medicinal, food	150 birr/kg
4	*Citrus limon*	Lomii	Medicinal, edible	150 birr/kg
5	*Coffea arabica*	Buna	Medicinal, drinking	400 birr/kg
6	*Coriandrum sativum*	Dimbilaala	Medicinal, spices	120 birr/kg
7	*Cucurbita pepo*	Dabaquula	Medicinal food	150 birr/kg
8	*Embelia schimperi*	Enqoqo	Medicinal, edible	100 birr/kg
9	*Lens culinaris*	Missira	Medicinal, food	300 birr/kg
10	*Linum usitatissimum*	Talbaa	Medicinal, edible	200 birr/kg
11	*Nigella sativa*	Tiqurazmud	Medicinal, spices	400 birr/kg
12	*Rhamnus prinoides*	Gesho	Medicinal, beverages	100 birr/kg
13	*Zingiber officinale*	Zinjibuilaa	Medicinal, spices	100 birr/kg

^*∗*^1$ = 55 Birr.

**Table 7:** Ranking of threats to medicinal plants cited by 11 key respondents (values 1–6: 1 = the least destructive and 6 = the most destructive).

Medicinal plant	Respondents (*R*_1_–*R*_11_)											Total	Rank
	*R*_1_	*R*_2_	*R*_3_	*R*_4_	*R*_5_	*R*_6_	*R*_7_	*R*_8_	*R*_9_	*R*_10_	*R*_11_		
Agricultural expansion	5	6	6	5	3	6	5	4	5	4	5	54	1^st^
Overgrazing	4	5	4	4	5	4	2	5	6	5	4	48	2^nd^
Drought	3	5	3	6	5	2	5	5	4	3	5	46	3^rd^
Deforestation	5	4	5	3	4	5	3	6	3	3	3	44	4^th^
Firewood collection	4	1	1	1	3	3	3	3	2	3	2	26	5^th^
Construction use	3	1	2	2	3	2	2	1	1	1	2	20	6^th^

## Data Availability

All data collected and analyzed are included in this published article and as Supplementary Materials.

## Supplementary Materials

Supplementary Materials Additional File 1: List of medicinal plants used by the local community in Ameya district to treat human and livestock ailments. The additional file shows the local names of the plants, disease treated, growth form, part used, modes of preparation, dosage, and voucher number.
