# Supplementary material for: Ethnobotanical Study on Medicinal Plants Used by the Local Communities of Ameya District, Oromia Regional State, Ethiopia
Source: Evid Based Complement Alternat Med. 2023 Dec 1;2023:5961067. doi: 10.1155/2023/5961067 (PMC10708956; doi:10.1155/2023/5961067)
Supplement: Supplementary Materials — Additional File 1: List of medicinal plants used by the local community in Ameya district to treat human and livestock ailments. The additional file shows the local names of the plants, disease treated, growth form, part used, modes of preparation, dosage, and voucher number. [file 5961067.f1.doc]

**Appendix 1**. List of medicinal plants used by the local community in Amaye District to treat human and livestock ailments

**Growth form** **(GF):** Herb-H, Shrub-SH, Tree-T: **Part used (PU)**: Bark-Ba, Fruit-Fr, Flower-Fl, Leaf-L, Latex-La, Stem-St, Seed-S, Bulb-Bu, Shoot-Sh, **ROA of Application –** MOA, **Used For (UF):** H-Human; L- Livestock; LH-Livestock and Human; **VN**- Voucher Number

| **No.** | **Scientific name** | **Family** | **Vernacular name** | **GF** | **Disease treated** | **PU** | **Mode of preparation and dosage** | **ROA** | **UF** | **VN** |
| --- | --- | --- | --- | --- | --- | --- | --- | --- | --- | --- |
| 1 | *Acmella caulirhiza* Del. | Asteraceae | Guutichaa | H | Tonsillitis | Fl | Fresh flowers chewed and swallowed | Oral | H | TT61 |
| 2 | *Afrocarpus falcatus*(Thunb.) C.N. | Podocarpaceae | Birbirsa | T | Snake bite | L | Crushed together with leaves of *Impatiens* sp. and mix with water drink it until recovery and also apply it on the affected part | Oral, dermal | L | TT33 |
| 3 | *Allium cepa* L*.* | Amaryllidaceae | Shunkurtii  diimaa | H | Poisoning | Bu | Powdered, mix with water and paint on the affected part | Dermal | LH | TT12 |
| 4 | *Allium sativum* L. | Amaryllidaceae | Qullubbiiadii | H | Common Cold | Bu | Bulb is crushed, mixed with honey, 2-3 teaspoon is eaten every day for five days | Oral | H | TT6 |
| 5 | *Aloe macrocarpa* Tod*.* | Asphodelaceae | Argiisa | H | Nose bleeding | L | Fresh leaves are crushed, squeeze the juice and add drops into the nose. This helps to stop nose bleeding. | Nasal | H | TT11 |
| 6 | *Aloe* sp. | Asphodelaceae | Eret | H | Wound | L | Use fire to warm the leaf, squeeze the juice, apply on the affected area 3 times for 2 days | Dermal | L | TT24 |
| 7 | *Artemisia abyssinica* Sch. Bip. ex A.Rich. | Asteraceae | Ariti | H | Evil spirit | R | Fresh root crushed and homogenized in water, then smell and creamed on skin | Dermal /nasal | H | TT76 |
| 8 | *Asparagus africanus* Lam. | Asparagaceae | Sariitii | SH | Spider poison | L | Fresh leaves crushed and applied/rubbed on the affected part | Dermal | H | TT58 |
| 9 | *Barleria eranthemoides* R. Br. ex C.B. Clarke | Acanthaceae | Shabi | H | Heart burn | R | The roots (length of finger) crushed and mixed with water and then drink | Oral | H | TT23 |
| 10 | *Brassica carinata*  A.Braun. | Brassicaceae | Gomenzer, Raafuu | H | ‘Lemtse’ | S | Powdered and mixed with honey, apply/paint on the affected part for 3 days | Dermal | H | TT36 |
|  |  |  |  |  | Bloating | L | Macerating in water, drink a cup once | Oral | L | TT68 |
| 11 | *Buddleja polystachya* Fresen. | Scrophulariaceae | Hanafaaree | T | Eye disease | L | Leaf is chewed and spitted on cattle eyes , apply two drops into the eyes | Optical | L | TT66 |
| 12 | *Calpurnia aurea* (Aiton) Benth. | Fabaceae | Ceekataa | SH | Stomachache | L | Fresh leaves is chewed and swallowed | Oral | L | TT22 |
| 13 | *Capparis sepiaria* L. | Capparaceae | Gursama | H | Stomachache | Ba | The bark is crushed and drink it with hot water, add 3-5 spoons | Oral | H | TT30 |
| 14 | *Capparis tomentosa* Lam*.* | Capparaceae | Harangamaa | SH | Toothache | L | The fresh leaf chewed and hold on the teeth for 2-3 hours | Oral | H | TT21 |
| 15 | *Carica papaya* L. | Caricaceae | Paappaayyaa | T | Malaria | L | The yellow leaves dried, powdered and boiled in water, drink a cup for 5 days. | Oral | H | TT34 |
| 16 | *Citrus limon* L.Osbeck | Rutaceae | Lomii | SH | Stomachache | Fr | Crushed together with *Allium sativum* and mixed with honey and water, take 1 tea cup of the mixture juice once | Oral | LH | TT9 |
| 17 | *Citrus* ^x^*aurantium* L. | Rutaceae | Orange | S | Stomachache | Fr | Powder the peel then mix with local alcohol, drink ½ a glass once | Oral | H | TT45 |
| 18 | *Coffea arabica* L*.* | Rubiaceae | Buna | SH | Wound | S | Roasted fresh /dried seeds grounded, and tied on the wound. for 2 hrs for 2 days | Dermal | H | TT14 |
| 19 | *Cordia africana* Lam. | Boraginaceae | Waddeessa | T | Bleeding | Ba | Fresh or dried bark crushed and mix with roasted coffee powder, boil then drink one coffee cup for four consecutive days | Oral | LH | TT8 |
| 20 | *Coriandrum sativum* L*.* | Apiaceae | Dimbilaala | H | Cutaneous leshmaniasis | L | Crushed with leaf of *Croton macrostachyus,* squeeze the water and apply on the affected area for 2-3 days | Dermal | H | TT2 |
| 21 | *Crinum abyssinicum* Hochst. ex A. Rich. | Amaryllidaceae | Yejibshenkrt | H | Ear disease | R | Crushed, homogenized in water and squeeze the juice, pour a drop into the ears for 3 days | Ear | H | TT78 |
| 22 | *Croton macrostachyus* Hochst. ex Del*.* | Euphorbiaceae | Bakkannisa | T | Wound | L,  St | Powdered mixed with water and butter and filtered, apply on the affected part | Dermal | H | TT49 |
| 23 | *Cucumis ficifolius* A. Rich. | Cucurbitaceae | Yemidir embuay | H | Stomachache | R | Crushed, homogenized in water and squeeze the juice, 1/3 tea cup is taken | Oral | L | TT64 |
| 24 | *Cucurbita pepo* L. | Cucurbitaceae | Dabaquula | SH | Bloat | R | Fresh root crushed and mixed with local alcohol drink ‘katikala’, drinking 2 tea cups for once | Oral | L | TT13 |
| 25 | *Cyathula polycephala* Bak. | Amaranthaceae | Chegogot | H | Skin rash (chiffee) | L | Crushed and squeeze the juice, apply on the affected part until recovery | Dermal | H | TT38 |
| 26 | *Cynodon nlemfuensis* Vanderyst. | Poaceae | Coqorsa  gurraacha | H | Tonsillitis | St | Chewed, gargle and spit | Topical | H | TT48 |
| 27 | *Datura stramonium* Thunb. | Solanaceae | Manjii | H | Dandruff (foroforii) | Fr | Dried and powdered then mixed with water and used to wash head skin. | Dermal | H | TT55 |
| 28 | *Dodonaea viscosa* Jacq. | Sapindaceae | Ittacha | SH | Wound | L | Dried leaves powdered, mixed with water and paint on the wound | Dermal | L | TT47 |
| 29 | *Dovyalis abyssinica* (A.Rich.) Warb. | Salicaceae | Koshime | SH | Intestinal parasites | Fr | Eat the fresh fruit in every morning on empty stomach | Oral | H | TT77 |
| 30 | *Echinops kebericho* Mesfin | Asteraceae | Qarabicho | H | Internal parasite | R | Dried root powdered and mixed with water then drink half a tea cup | Oral | L | TT40 |
| 31 | *Ehretia cymosa* Thonn*.* | Boraginaceae | Ulaagaa | T | Toothache | L | Fresh leaves chewed, hold on the teeth | Oral | H | TT71 |
| 32 | *Embelia schimperi* Vatke. | Primulaceae | Enqoqo | SH | Tape worm | Fr | Powdered dried fruit is homogenized in water and drink one glass | Oral | H | TT60 |
| 33 | *Eucalyptus globulus* Labill. | Myrtaceae | Baargamoo | T | Cough | L | Fresh young leaves are boiled, fumigate and inhale the vapor under sealed clothes at evening time | Nasal,  topical | H | TT31 |
| 34 | *Euphorbia tirucalli* L. | Euphorbiaceae | Knchib | SH | Skin disease | La | Latex/sap is collected and applied on the affected area | Dermal | H | TT57 |
| 35 | *Ficus sycomorus* L. | Moraceae | Odaa | T | Hepatitis | Ba | Latex/sap is collected and applied on the affected area | Dermal | H | TT65 |
| 36 | *Gossypium herbaceum* L. | Malvaceae | Yetiti fire | SH | Eye infection | S | Dry seeds are chewed then put a tiny drop/piece into the eyes | optical | L | TT32 |
| 37 | *Grewia ferruginea Hochst.* ex. A. Rich. | Malvaceae | Dhoqonuu | SH | Retained placenta | La | Latex crushed, mixed with water , a glass is given | Oral | L | TT53 |
| 38 | *Guizotia scabra* (Vios) Chiov. | Asteraceae | Tuufoo | H | External parasite | L | Fresh leaves crushed and applied on the affected part | Dermal | H | TT81 |
| 39 | *Guizotia schimperi* Sch. Bip, ex Walp. | Asteraceae | Adaa | H | Eye infection | Fl | Infuse the fresh flower, squeeze the juice then apply a drop into the eyes for two days | optical | H | TT67 |
| 40 | *Gymnanthemum amygdalinum*(Delile) Sch.Bip. | Asteraceae | Gerawa | SH | Dandruff, malaria, heart problem | L, R | Crushed and apply the paste; Squeeze the juice homogenize with water then drink half a liter once | Oral, dermal | H | TT4 |
| 41 | *Gymnanthemum auriculiferum*(Hiern) Isawumi | Asteraceae | Reejii | SH | Wound | L | Squeeze the juice from the fresh leaves, then apply on the affected part | Dermal | H | TT75 |
| 42 | *Hagenia abyssinica* (Brue) (J. F. Gmel) | Rosaceae | Koso | T | Tape worm and diarrhea | L | Crushed leaves mixed with local drink ’tella’ then take half a litter at once | Oral | H | TT3 |
| 43 | *Indigofera tinctoria* L. | Fabaceae | Dingetenya | H | Sudden sickness | R | Chopped root mixed with salt, and given to the cattle. | Oral | L | TT41 |
| 44 | *Jatropha curcas* L. | Euphorbiaceae | Abatamuluk | SH | Intestinal worm | R | Root is crushed, homogenized in water and drink half of coffee cup | Oral | H | TT20 |
| 45 | *Juniperus procera* Hochst. ex Endl. | Cupressaceae | Tsed | T | Ear disease | S | The seed is crushed mixed with little water then filter and put 3-7 drops into the ears | Ear | H | TT19 |
| 46 | *Justicia schimperiana* T.Anderson. | Acanthaceae | Sensel | SH | Bloating | L | Leaves are crushed then mixed with water, drenched then given without filtering | Oral | L | TT17 |
| 47 | *Lens culinaris* Medik. | Fabaceae | Missira | H | Spider poisoning | S | Chewing, and apply mouthful of paste once | Oral | H | TT69 |
| 48 | *Leonotis ocymifolia* (Burm,f.) Iwarsson | Lamiaceae | Raaskimmirii | H | Febrile illness | L | Crushed leaves, homogenized with water and one glass is taken until recovery | Oral | H | TT63 |
| 49 | *Lepidium sativum* L. | Brassicaceae | Feto, Shinfaa | H | Gastrointestinal disorder | S | Dried seed powder mixed with water then drink a tea cup for 2 days | Oral | LH | TT37 |
|  |  |  |  |  | Malaria | S | Dried seed powdered and eaten with ‘injera’ to get cure from malaria. Rubbed the body as a protection | Oral | H | TT35 |
| 50 | *Leucas martinicensis* (Jacq) R. Br. | Lamiaceae | Fidoo | H | Eye disease | St | Burn the stem for a smoke and let the patient laid for the smoke towards the affected part | Optical | H | TT52 |
| 51 | *Linum usitatissimum* L. | Linaceae | Talbaa | H | Retention of placenta | R | Boil the root and half a liter is given | Oral | L | TT5 |
| 52 | *Nicotiana tabacum* L. | Solanaceae | Timbaho | H | Cough and leeches | L | Crushed, homogenized in water and half a liter is given | Oral | L | TT1 |
| 53 | *Nigella sativa* L. | Ranunculaceae | Tiqurazmud | H | Common cold, asthma | S | Pounded and sniff the powder smell for 3-4 times per day. | Nasal/Oral |  | TT39 |
| 54 | *Ocimum gratissimum* L. | Lamiaceae | Damakesie | SH | Skin | L | Crushed, homogenized in water and drink half a tea cup once | Rubbing and oral | H | TT7 |
| 55 | *Ocimum gratissimum* subsp. *gratissimum* | Lamiaceae | Ancabbii | SH | Febrile illness | L | Crushed, homogenized in water and drink a glass until recovery | Oral | H | TT44 |
| 56 | *Olea europaea* subsp. *cuspidata* (Wall. & G.Don) Cif | Oleaceae | Ejersa | T | Itchy skin | L | Fresh leaf is boiled and steam the affected part with the vapor. | Dermal | H | TT10 |
| 57 | *Phoenix reclinata* Jacq. | Arecaceae | Meexxi | T | Eye disease | L , St | Leaf and stem chewed together and put two drops into the eyes | Optical | L | TT54 |
| 58 | *Phragmanthera macrosolen* (Steud. ex A.Rich) M.G.Gilbert | Loranthaceae | Dagaluuceakaa | Ep | Stomach ache | L | Leaf is pounded and mixed with water or local drink ‘tella’. | Oral | L | TT72 |
| 59 | *Phragmites australis*(Cav.) Steud. | Poaceae | Shenbeko | H | Rh factors ‘’shotelay” | R | Piece of the root is tied on the neck | Neck/  Topical | H | TT46 |
| 60 | *Phytolacca americana*L. | Phytolaccaceae | Endod | S | Rabies | R | Crushed fresh root is mixed with milk and drink a glass for 2 days | Oral | H | TT74 |
| 61 | *Premna resinosa* Schauer | Lamiaceae | Urggesaa | T | Tooth infection | R | Chew the root and paste on the affected part for 2 days | Oral | H | TT73 |
| 62 | *Prunus africana* (Hook.f.) Kalkman | Rosaceae | Hoomii | T | Wound | Ba | Powdered and added directly on the wound (of donkey, mule and horse) | Dermal | L | TT62 |
| 63 | *Pycnostachys abyssinica* Fresen. | Lamiaceae | Yeeroo | H | Eye disease | L | Squeeze the juice from the fresh leaves and apply 2 drops into the eyes | Optical | LH | TT50 |
| 64 | *Rhamnus prinoides* L'Hér. | Rhamnaceae | Gesho | SH | Liver problem | L | Powdered, mixed with honey and eaten for 3 days before food. | Oral | H | TT79 |
| 65 | *Ricinus communis* L. | Euphorbiaceae | Gulo | H | Amoebiasis | S | Chewing the dried seed | oral | H | TT59 |
| 66 | *Ritchiea albersii* Gilg*.* | Capparaceae | Arbuu | T | Cough | S | Crushed and mixed with tea/water then drink 1/2 tea cup | Oral | H | TT70 |
| 67 | *Rosa abyssinica* R. Br. | Rosaceae | Qinidaabii | H | Stomach pain | R | Fresh root chewed and take 2 tea spoon of the the juice | Oral | H | TT15 |
| 68 | *Rumex nepalensis* Spreng. | Polygonaceae | Tultii | H | Spider poison | L | Rubbed on affected skin. | Dermal | H | TT43 |
| 69 | *Ruta chalepensis*L. | Rutaceae | Cilaattama | H | Stomachache | L | Leaf crushed and mix with one cup of local alcohol drink ‘Areki’ then take a cup | Oral | L | TT18 |
| 70 | *Schinus molle* L. | Anacardiaceae | Qundbarbare | T | Tonsillitis and bile, | S, L | The seed (3-5 seeds) powdered and boiled then 1 cup is given in the mornings; the leaves (5) are crushed and the filter is given for 5 days | Oral | H | TT16 |
| 71 | *Senegalia senegal* (L.) Britton | Fabaceae | Saphansa | T | Fire wound | La | 1-2 bean size solid latex is powdered latex is applied on the wound | Dermal | LH | TT25 |
| 72 | *Stereospermum kunthianum* Cham*.* | Bignoniaceae | Botoroo | T | Snake bite | R, L | Dried Root and leaves powdered and mixed with water and drink one cup | Oral | LH | TT51 |
| 73 | *Trachyspermum ammi* Sprague | Apiaceae | Nechazmud | H | Urine retention | S | Seed crushed and mixed with water and drink a glass | Oral | H | TT42 |
| 74 | *Tragia plukenetii* Radcl.-Sm. | Euphorbiaceae | Sama | H | Myiasis | L | Crush the fresh leaves and homogenize with water then drink a tea cup | Oral | H | TT56 |
| 75 | *Vachellia abyssinica* (Hochst. ex Benth.) Kyal.&Boatwr. | Fabaceae | Laaftoo | T | Horse scabies | R,  Ba | Fresh root and bark grounded together, mix with water and wash the affected part with the solution | Dermal | L | TT27 |
| 76 | *Vachellia etbaica* (Schweinf.) Kyal.&Boatwr. | Fabaceae | Doddota | T | Internal parasite | Fr | Crush the fruit with sugar and honey, drink 3 to 4 cup at once | Oral | H | TT26 |
| 77 | *Withania somnifera* (L.) Dunal | Solanaceae | Kumo | SH | Malaria | L | Dried leaf powdered homogenized with water, then drink a tea cup for 4 days | Oral | H | TT29 |
| 78 | *Zingiber offcinale* Roscoe | Zingiberaceae | Zinjibuilaa | H | Cough, common cold & tonsillitis | R | The roots crushed and boiled with tea and then taken. | Oral | H | TT28 |
